# Evaluation of Curcumin as a Supplement to Improve Mitotane Effects on Adrenocortical Carcinoma

**DOI:** 10.3390/cells15161498

**Published:** 2026-08-20

**Authors:** Marta Claudia Nocito, Alice Amico, Tarig Hamad, Alessandro Cormace, Constanze Hantel, Catia Morelli, Diego Sisci, Marilena Lanzino, Vincenzo Pezzi, Ivan Casaburi, Rosa Sirianni

**Affiliations:** 1 Department of Pharmacy, Health and Nutritional Sciences, University of Calabria, 87036 Rende, CS, Italy; martaclaudia.nocito@unical.it (M.C.N.); alice.amico@unical.it (A.A.); tarig.hamad@unical.it (T.H.); alessandro.cormace@unical.it (A.C.); vincenzo.pezzi@unical.it (V.P.); 2 Department of Endocrinology, Diabetology and Clinical Nutrition, University Hospital Zurich (USZ), University of Zurich (UZH), 8091 Zürich, Switzerland; constanze.hantel@usz.ch; 3 Centro Sanitario, Department of Pharmacy, Health and Nutritional Sciences, University of Calabria, Ponte P. Bucci, 87036 Rende (CS), Italy; catia.morelli@unical.it (C.M.); diego.sisci@unical.it (D.S.); marilena.lanzino@unical.it (M.L.)

**Keywords:** curcumin, adrenocortical carcinoma, cell metabolism, mitotane, antioxidant mechanism, NRF2, steroidogenic enzymes, SF-1

## Abstract

For many types of cancer, pre-clinical studies have shown that dietary interventions and supplements can be effective in reducing the toxicity and increasing the efficacy of chemotherapeutics. In this context, the polyphenol curcumin is an attractive molecule. We have previously demonstrated that curcumin inhibits adrenocortical carcinoma (ACC) cell growth, has an impact on ACC cell metabolism, decreasing cholesterol availability and promoting glucose and glutamine metabolism. In this study, we evidenced that curcumin downregulates the transcription factors SF-1 and SREBPs and their targets, while inducing a ROS-dependent, HIF1α- and NRF2-mediated metabolic rewiring. NRF2 sustained an adaptive antioxidant mechanism dependent on glutamine, cysteine, and glycine uptake to support glutathione synthesis and avoid lipid peroxidation. Furthermore, the combination of curcumin with mitotane demonstrated synergistic effects in inhibiting ACC cell viability and reducing steroidogenic gene expression. These synergistic effects were observed with sub-therapeutic doses of mitotane, which are reached by patients who fail to attain the therapeutic plasma concentrations of the drug. Crucially, in vivo, curcumin administration to tumor-free mice significantly upregulated NRF2 expression and preserved liver tissue integrity. These results warrant further preclinical evaluation of the proposed combination therapy, particularly for those patients who fail to achieve or maintain mitotane plasma concentrations in the therapeutic range.

## 1. Introduction

Adrenocortical carcinoma (ACC) is an aggressive cancer with very limited therapeutic options. Mitotane, an adrenolytic agent that inhibits steroidogenesis and promotes apoptosis, remains the cornerstone of ACC treatment. In patients with advanced, metastatic, or unresectable disease, the standard first-line approach combines mitotane with EDP chemotherapy (etoposide, doxorubicin, cisplatin plus mitotane; EDP-M). Nevertheless, most patients eventually develop therapeutic resistance, and the prognosis remains extremely poor, with a 5-year overall survival rate below 15% [1]. Despite significant efforts, novel therapeutic strategies, including targeted therapies and immune checkpoint inhibitors, have failed to provide consistent clinical benefits in ACC patients [2], further emphasizing the continued central role of mitotane in ACC management. 

A major limitation for mitotane is the prolonged time required to achieve the therapeutic plasma concentration. Treatment efficacy is generally associated with plasma concentration above 14 mg/L but necessitates careful therapeutic monitoring [2] since mitotane causes several adverse effects; severe neuropsychological toxicity is observed at concentrations exceeding 20 mg/L, although lower concentrations also impair patients’ quality of life. In addition, mitotane frequently alters many laboratory parameters, including thyroid tests, gonadal tests, and lipid profiles, and patients frequently require hormonal replacement therapy and statin administration. Gastrointestinal symptoms, severe tiredness, dizziness, skin rashes, and hepatotoxicity are also common and frequently lead to treatment discontinuation.

For many types of cancer, pre-clinical studies have shown that dietary interventions, such as caloric restriction, intermittent fasting, and ketogenic diets, are effective in reducing the toxicity and increasing the efficacy of chemotherapeutics [3]. Additionally, dietary interventions have been shown to reduce cancer growth, progression, and metastasis and are promising for improving cancer outcomes in early-phase clinical studies. For ACC, the benefits of specific diets or the inclusion of nutraceutical supplements to improve the response to conventional therapies, i.e., mitotane, remain largely unexplored.

Curcumin, a naturally occurring polyphenolic phytoalexin, has attracted a lot of attention because of its wide range of biological activities, including antioxidant, anti-inflammatory, antimicrobial, antiviral, and anticancer [4], with the latter depending on its ability to activate different cell death mechanisms [5]. We have proved that curcumin reduces growth and migration and induces apoptosis of ACC cells, while reprograms ACC cell metabolism, promoting a glycolytic phenotype [6]. Importantly, curcumin reduces the expression of hydroxymethylglutaryl-CoA reductase (HMGCR), the rate-limiting enzyme in cholesterol synthesis, which we have shown to be upregulated by mitotane [7].

Transcription of cholesterol synthesis enzymes is regulated by the transcription factor sterol regulatory element-binding protein 2 (SREBP-2); however, in adrenal cells, this process is also driven by the nuclear receptor steroidogenic factor-1 (SF-1) [8]. SF-1, which is primarily known as a key factor for steroidogenic enzymes [9], sustains the expression of glycolytic enzymes [10] and represents a valuable diagnostic biomarker for ACC [11]. The observation that curcumin simultaneously reduces HMGCR while increasing glycolytic gene expression suggests a fine-tuning of the transcriptional machinery controlling metabolic and steroidogenic pathways. Ultimately, the rationale for exploring curcumin adjunct therapy lies in its potential to mitigate mitotane-induced hypercholesterolemia. While mitotane elevates lipid levels, curcumin exerts a therapeutic systemic lipid-lowering effect by reducing total cholesterol, LDL, and triglycerides [12]. Then, curcumin holds the potential of enhancing the antitumor effects of mitotane, while mitigating some of its unwanted metabolic side effects.

Based on these considerations, the present study aimed to deepen the understanding of the molecular mechanisms activated by curcumin in ACC cells, and to evaluate, in a preclinical setting, the benefits of curcumin supplementation in combination with mitotane.

## 2. Materials and Methods

### 2.1. Cell Cultures

H295R cells were obtained from the American Type Culture Collection (ATCC, Rockville, MD, USA). MUC-1 cells were obtained from Prof. Constanze Hantel (University of Zurich, Switzerland) [13]. All of the cell lines used were grown in a humidified 5% CO_2_ incubator at 37 °C and cultured as previously described [6]. Specific treatment conditions, including exposure times and concentrations, are detailed within each experimental method section. Treatments were added to complete medium [6].

### 2.2. RNA Extraction, Reverse Transcription and Real-Time PCR

Total RNA was isolated using the TRIzol reagent (Invitrogen, Carlsbad, CA, USA). One microgram of total RNA was reverse transcribed into a final volume of 50 μL using the High Capacity cDNA Reverse Transcription Kit (Thermo Fisher, Foster City, CA, USA). cDNA was diluted 1:3 in nuclease-free water and subsequently used for real-time PCR analysis. Primer sequences are reported in Table A1. PCR amplification was carried out in the QuantStudio™ 3, Real-Time PCR System (Thermo Fisher) using 0.2 μM of each primer. PowerUp™ SYBR™ Green Master Mix (Thermo Fisher) with the dissociation protocol was used for gene amplification; negative controls were prepared using water in place of first-strand cDNA. Target gene expression levels were normalized to 18S rRNA (18S), and relative quantification was determined using the ΔΔCt method, with results expressed as fold-changes relative to the calibrator sample. Each experiment was performed in duplicate and independently replicated three times.

### 2.3. Determination of Oxidative Stress

Oxidative stress levels were assessed using the Muse Oxidative Stress Kit from Luminex (Aurogene, Italy). H295R and MUC-1 cells were seeded in 6-well plates containing growth medium, and then treated with Curcumin (10 and 20 µM) or MitoPQ (1 μM) for the appropriate time. After the treatment period, the cells were harvested by trypsinization, counted, and resuspended in 1 mL of 1X Assay Buffer to a final density of 1 × 10^6^ cells. For each sample, 10 µL of cells (~1 × 10^4^) were mixed with 190 µL of Muse Oxidative Stress Reagent (working solution) in a test tube and incubated for 30 min at 37 °C. Oxidative stress was then analyzed using the Guava^®^ Muse^®^ Cell Analyzer (Luminex), acquiring a total of 5000 events per sample. Cells treated with 3 mM H_2_O_2_ for 2 h were used as a positive control. Each experiment was performed with six technical replicates and independently repeated three times.

### 2.4. Lipid Peroxidation

H295R and MUC-1 cells were seeded onto glass coverslips placed inside six-well plates. After 48 h, cells were treated for an additional 48 h with Curcumin (20 μM). Following the treatment, cells were washed twice with PBS and incubated with 5 μM BODIPY™ 581/591 C11 for 20 min at 37 °C. Cells were then washed twice with PBS and fixed with 4% paraformaldehyde (PFA) for 15 min at room temperature. Finally, coverslips were mounted onto glass slides and fluorescence signals were visualized using an FV3000 confocal laser scanning microscope (Olympus Corporation, Tokyo, Japan).

### 2.5. Glutathione Determination

Total glutathione (GSH) levels were quantified using the Glutathione Colorimetric Detection Kit (Invitrogen). Briefly, H295R cells were seeded in 6-well plates and exposed to Curcumin 20 µM for 48 h. Following treatments, cells (1 × 10^6^) were harvested by trypsinization and resuspended in 1 mL of an ice-cold 5% aqueous solution of 5-sulfo-salicylic acid dihydrate (SSA). Cells were lysed via vigorous vortexing, then the samples were incubated for 10 min at 4 °C, and centrifuged at 14,000 rpm for 10 min at 4 °C. The resulting supernatants were diluted 1:4 (*v*/*v*) with 1X Assay Buffer. Cell extracts were then incubated with the Reaction Mixture for 20 min, and total GSH content was detected according to the manufacturer’s instructions. Absorbance was recorded at 405 nm using a Synergy H1 plate reader (BioTek Instruments, Inc., Winooski, VT, USA). Each experiment was performed with six technical replicates and independently repeated three times.

### 2.6. Western Blot Analysis

Total protein was extracted using RIPA buffer containing 50 mM Tris-HCl, 150 mM NaCl, 1% NP-40, 0.5% sodium deoxycholate, 2 mM sodium fluoride, 2 mM EDTA, 0.1% SDS, and a mixture of protease inhibitors. Protein concentrations were determined using the Bradford assay (BIO-RAD), and equal amounts of protein were subjected to SDS-PAGE-followed by electrotransfer onto a nitrocellulose membrane. Membranes were incubated overnight at 4 °C with anti-phosporylated ERK1/2 Antibody (pERK1/2; sc-7383; 1:1000; Santa Cruz Biotechnology); anti-ERK2 Antibody (sc-1647; 1:1000; Santa Cruz Biotechnology). After washing, membranes were incubated for 1 h at room temperature with appropriate horseradish peroxidase (HRP)-conjugated secondary antibodies. Immunoreactive bands were visualized using the enhanced chemiluminescence (ECL) detection system (Santa Cruz Biotechnology, sc-2048) and imaged with the iBright detection system (Thermo Fisher). Band intensities were quantified using Image Lab software (V6.0.0, Bio-Rad Laboratories, Hercules, CA, USA).

### 2.7. Immunofluorescence Staining

H295R cells were seeded onto coverslips placed in six-well plates and treated as reported. After treatment, cells were washed twice with PBS and fixed with 4% paraformaldehyde (PFA) for 15 min at room temperature. Fixed cells were washed twice with PBS and permeabilized with a solution of Triton X-100 in PBS (0.1% *v*/*v*) for 3 min, then blocked with 3% bovine serum albumin (BSA) in PBS for 30 min at room temperature in a humidified chamber. Cells were then incubated overnight at 4 °C with an anti-NRF2 (PA5-88084; Invitrogen) primary antibody, diluted 1:100 in PBS. Following two washes with PBS, cells were incubated for 1 h at room temperature with the appropriate secondary antibody diluted 1:1000 in PBS. After two additional PBS washes, nuclei were counterstained with DAPI (0.2 µg/mL in PBS) for 5 min at room temperature. Coverslips were washed twice with PBS, mounted onto glass slides, and examined using an FV3000 confocal laser scanning microscope (Olympus Corporation, Tokyo, Japan).

### 2.8. Cell Viability Assay

Cell viability was assessed using the 3-(4,5-dimethylthiazol-2-yl)-2,5-diphenyltetrazolium bromide (MTT) colorimetric assay (Sigma-Aldrich). H295R cells were seeded in 48-well plates and allowed to adhere for 48 h before treatment. Cells were then exposed for 48 h to increasing concentrations of curcumin (0, 10, or 20 µM; Sigma-Aldrich C1386) dissolved in DMSO (final concentration ≤ 0.001% *v*/*v*) in the presence or absence of mitotane (0, 10, 20, or 40 µM; Sigma-Aldrich SML1885). Following treatment, freshly prepared MTT solution in PBS was added to each well to achieve a final concentration of 0.33 mg/mL, and cells were incubated for 2 h at 37 °C in a humidified incubator with 5% CO_2_. The medium was then removed, and the resulting formazan crystals were dissolved in 200 µL of DMSO (Sigma-Aldrich) under gentle agitation. Absorbance was measured at 570 nm using a Synergy H1 plate reader (BioTek Instruments, Inc., Winooski, VT, USA). Each experimental condition was tested in six technical replicates, and all experiments were independently repeated three times.

### 2.9. Synergy Treatment Analysis

Drug synergy between curcumin and mitotane was evaluated in H295R cells by measuring cell viability using the MTT colorimetric assay. Drug-drug interactions were analyzed using the SynergyFinder+ (version 3.10.3) web tool (https://synergyfinder.org/), using four reference models: Zero Interaction Potency (ZIP), Bliss independence, Loewe additivity, and Highest Single Agent (HSA). To provide a comprehensive evaluation of the interaction, synergy scores were calculated using all four models. However, the HSA model was selected as the primary metric for interpreting synergistic effects [14], as it offers a conservative assessment based on the expected activity of the most effective single agent.

### 2.10. Animal Study

The animal study protocol was approved by the National Review Board (Ministero della Salute) (protocol code 139-2017-PR). Mice were housed in a temperature-controlled (22 ± 2 °C) and humidity-controlled (55 ± 10%) facility under a standard 12 h light/dark cycle. Animals had ad libitum access to a standard rodent pellet diet and water. They were kept in polycarbonate cages with sawdust bedding. Curcumin (500 mg/Kg) was administered daily by gavage to 8-week-old tumor-free female Foxn1nu mice (Harlan Envigo). Four weeks later, mice were euthanized, and livers were harvested, weighed, and used for further analyses.

### 2.11. Immunohistochemistry

Paraffin-embedded sections (5 μm) were mounted on slides pre-coated with poly-lysine, and then they were deparaffinized and dehydrated (seven to eight serial sections). Immunohistochemical experiments were performed using rabbit polyclonal NRF2 primary antibody (PA5-88084; Invitrogen) at 4 °C overnight. Then, a biotinylated goat-anti-rabbit IgG was applied for 1 h at room temperature, followed by an avidin-biotin-horseradish peroxidase reaction (Vector Laboratories, Newark, CA, USA). Immunoreactivity was visualized using the diaminobenzidine chromogen (Sigma-Aldrich). Counterstaining was carried out with Hematoxylin (Sigma-Aldrich). Hematoxylin and eosin Y staining was performed as suggested by the manufacturer (Bio-Optica, Milan, Italy). Formalin-fixed, paraffin-embedded liver sections were deparaffinized, rehydrated, and stained using a Masson’s trichrome kit (Sigma-Aldrich) according to the manufacturer’s protocol to evaluate liver fibrosis [15]. Briefly, collagen fibers were stained blue and cytoplasm red. Slides were dehydrated, cleared, mounted, and examined under a light microscope.

### 2.12. Statistical Analysis

All experiments were performed at least three independent times. Data are presented as the mean ± standard deviation (SD). Statistical analyses were performed using GraphPad Prism (v.5.0; GraphPad Software, Inc., San Diego, CA, USA). Differences among multiple groups were evaluated by one-way analysis of variance (ANOVA) followed by Bonferroni’s multiple comparison post hoc test. A *p* value < 0.05 was considered statistically significant.

## 3. Results

### 3.1. Curcumin Reduces Expression of SF-1 and Its Targets

**Figure 1:**
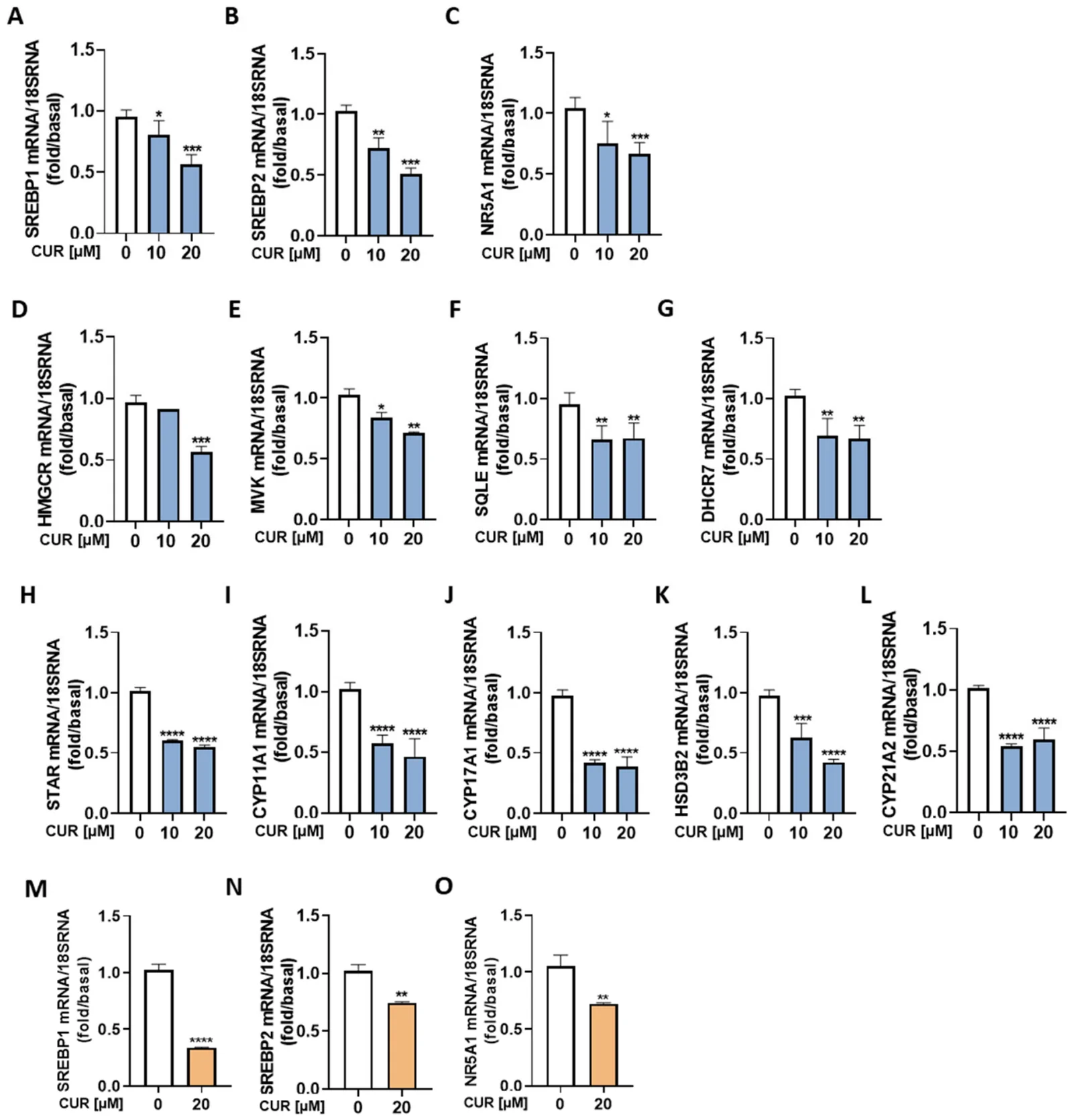
Curcumin decreases expression of SF-1 and its target genes. (**A**–**L**) H295R cells were treated for 48 h with curcumin at the indicated concentrations. Real time PCR was used to evaluate mRNA levels of *SREBP1*, *SREBP2*, *NR5A1*, and genes related to cholesterol and steroid synthesis. (**M**–**O**) mRNA expression of *SREBP1*, *SREBP2*, and *NR5A1* in MUC-1 cells treated for 48 h with curcumin (20 µM). Bars represent means ± SD of three independent RNA samples. * *p* < 0.05; ** *p* < 0.01; *** *p* < 0.001; **** *p* < 0.0001.

Our previous study demonstrated that curcumin reduces HMGCR expression, consequently lowering cholesterol levels. While cholesterol synthesis genes are typically regulated by sterol regulatory element-binding proteins (SREBPs), in steroidogenic cells—such as adrenocortical cells—their transcription can also be modulated by SF-1 [8]. RT-qPCR analysis revealed that curcumin down-regulates SREBP1, SREBP2, and NR5A1 (encoding Steroidogenic Factor-1, SF-1) in a dose-dependent manner (Figure 1A–C). Analysis of cholesterol synthesis genes confirmed the down-regulation of HMGCR (Figure 1D) and demonstrated a significant decrease in the mRNA levels of MVK, SQLE, and DHCR7 **(**Figure 1E–G). Similarly, curcumin suppressed the expression of key steroidogenesis-related genes, including *StAR*, *CYP11A1*, *CYP17A1*, *HSD3B2*, and *CYP21A1* (Figure 1H–L). Additionally, the mRNA levels of *SREBP1*, *SREBP2*, and *NR5A1* were also reduced in MUC-1 cells (Figure 1M–O).

### 3.2. Curcumin-Induced Expression of Glycolytic Genes Is ROS-Dependent

**Figure 2:**
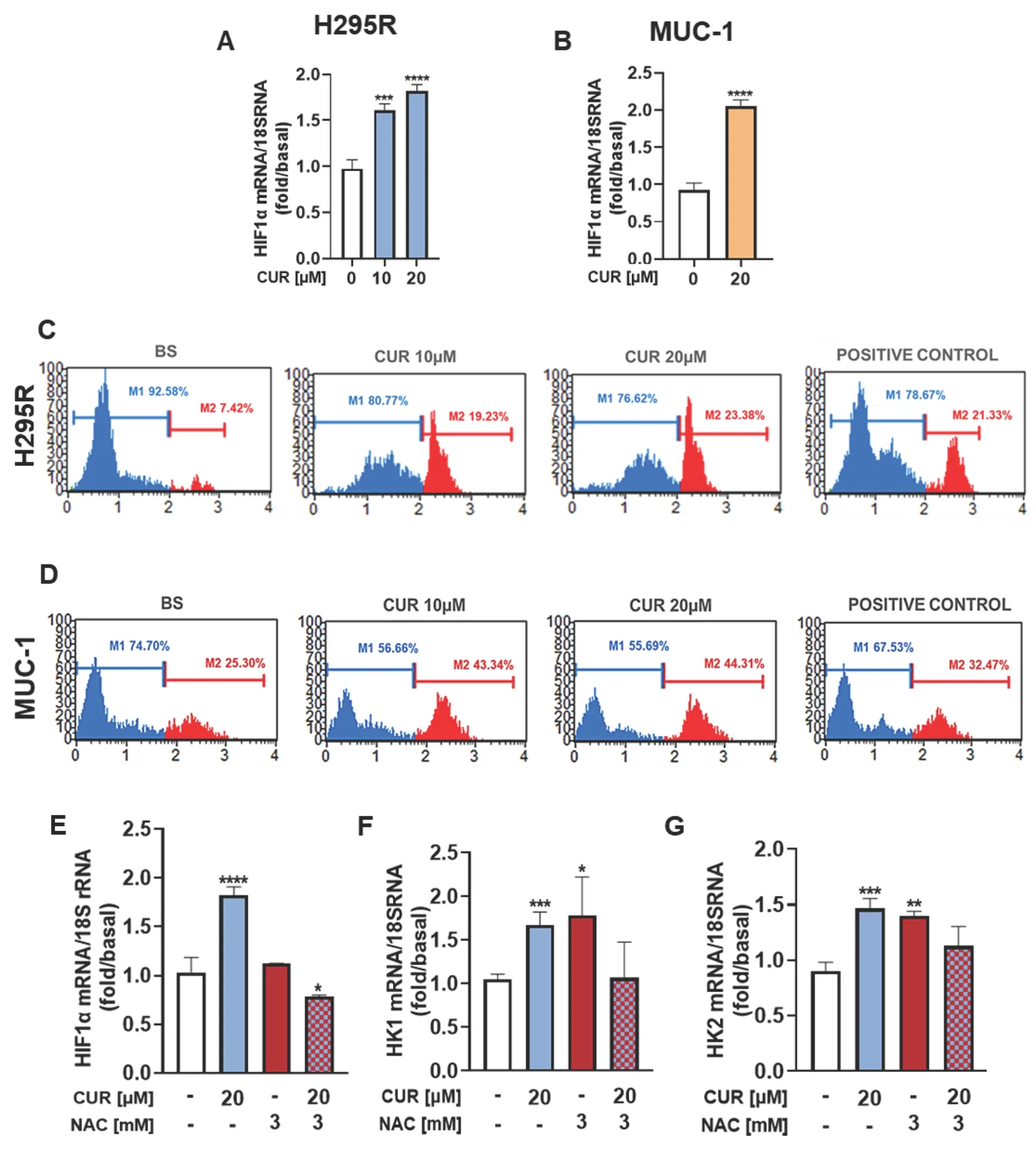
Curcumin increases ROS production to support *HIF1α* expression. (**A**,**B**) mRNA expression of *HIF1α* in ACC cell lines treated for 48 h with curcumin. (**C**,**D**) Flow cytometric analysis of reactive oxygen species (ROS) in ACC cells treated with curcumin for 48 h. Cells treated with H_2_O_2_ for 2 h were used as positive control. ROS-positive cells are shown in red. (**E**–**G**) mRNA expression of *HIF1α*, *HK1*, and *HK2* in H295R cell lines treated for 48 h with curcumin and N-acetylcysteine (NAC), as indicated. Bars represent means ± SD of three independent RNA samples; * *p* < 0.05; ** *p* < 0.01; *** *p* < 0.001; **** *p* < 0.0001 compared to untreated cells.

SF-1 is known to regulate glycolytic genes in adrenal cells [10]; therefore, our finding that curcumin down-regulates SF-1 would suggest a potential decrease in glycolysis. However, our previous study showed that curcumin actually increases glycolytic gene expression, implying the involvement of an alternative regulator. We first investigated Hypoxia-Inducible Factor 1-alpha (*HIF1α*) as a candidate. QPCR analysis showed that curcumin increases *HIF1α* expression in both H295R and MUC-1 cells (Figure 2A,B). *HIF1α* is upregulated by hypoxia, a condition that can be reached when cells rapidly consume oxygen and produce ROS. Accordingly, treatment with 10 and 20 µM curcumin increased ROS levels in H295R cells by 2.7- and 3.1-fold, respectively (Figure 2C). In MUC-1 cells, ROS levels rose by 1.76-fold in response to both doses of curcumin (Figure 2D). To prove the specific involvement of ROS in *HIF1α* regulation, we treated cells with the antioxidant N-acetylcysteine (NAC); this treatment successfully abrogated the curcumin-dependent effects on *HIF1α* (Figure 2E), and consequently, on *HK1* and *HK2* expression (Figure 2F,G).

### 3.3. Curcumin-Dependent Metabolic Rewiring Supports an Antioxidant Response

**Figure 3:**
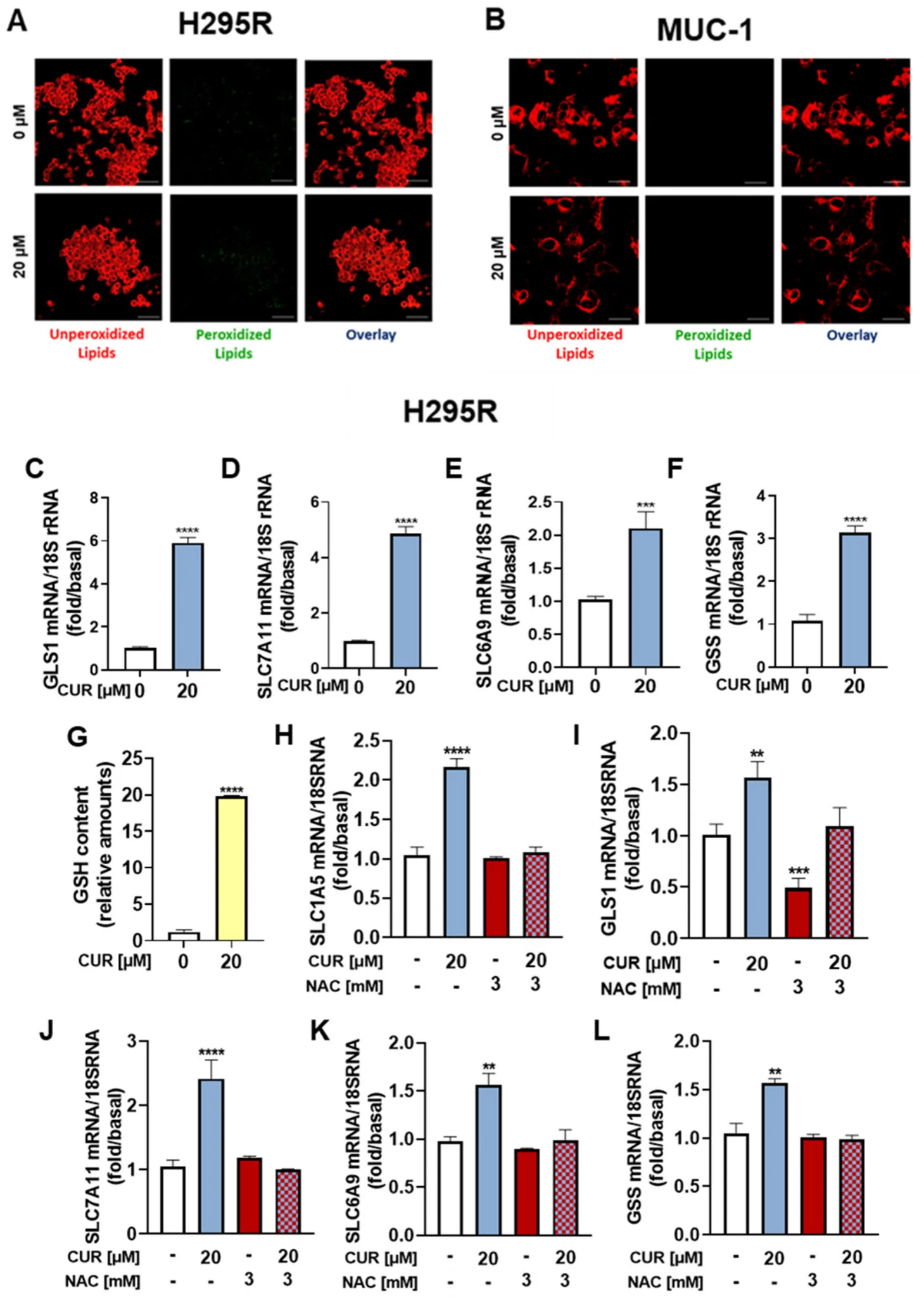
Curcumin activates an antioxidant response. (**A**,**B**) Confocal images of lipid peroxidation detected by BODIPY-C11 fluorescent dye after 48 h of treatment. Scale bar: 50 µm. (**C**–**F**) mRNA expression of GLS1, SLC7A11, SLC6A9 and GSS. (**G**) Glutathione (GSH) content normalized to the number of cells. (*n* = 3, means ± SD, **** *p* < 0.0001). (**H**–**L**) mRNA expression of genes related to the antioxidant mechanism in H295R cells treated for 48 h with curcumin and N-acetylcysteine (NAC), as indicated. Bars represent means ± SD of three independent RNA samples; ** *p* < 0.01; *** *p* < 0.001; **** *p* < 0.0001.

Given the rise in ROS levels, we investigated whether curcumin induces lipid peroxidation as part of its anticancer mechanism. C11-BODIPY staining revealed no evidence of lipid peroxidation in curcumin-treated H295R or MUC-1 cells (Figure 3A,B), suggesting that an efficient antioxidant mechanism had to be activated. We previously showed that curcumin induces ACC cells to rewire their metabolism toward glutamine (Gln) uptake [6]. Hypothesizing that Gln could support an antioxidant defense, we evaluated expression of the Gln-metabolizing enzyme glutaminase-1 (*GLS1*) and found it to be significantly upregulated (Figure 3C). GLS1 converts Gln into glutamate (Glu), one of the three amino acids building the tripeptide glutathione (GSH; Glu-Cys-Gly). This abundant intracellular antioxidant is known to be upregulated by curcumin [16]. Indeed, we found that curcumin increased the expression of *SLC7A11* and *SLC6A9*—transporters for cystine and glycine, respectively—in ACC cells (Figure 3D,E). Accordingly, the GSH synthesis enzyme glutathione synthase (GSS) was also upregulated (Figure 3F), leading to an elevation in total glutathione (GSH) levels) (Figure 3G).

To assess whether ROS drive this antioxidant defense mechanism, we utilized the scavenger NAC. Quenching ROS prevented the curcumin-dependent upregulation of antioxidant genes (Figure 3H–L), confirming the necessity of ROS activation.

### 3.4. The Transcription Factor NRF2 Is Involved in Curcumin-Induced Antioxidant Response

**Figure 4:**
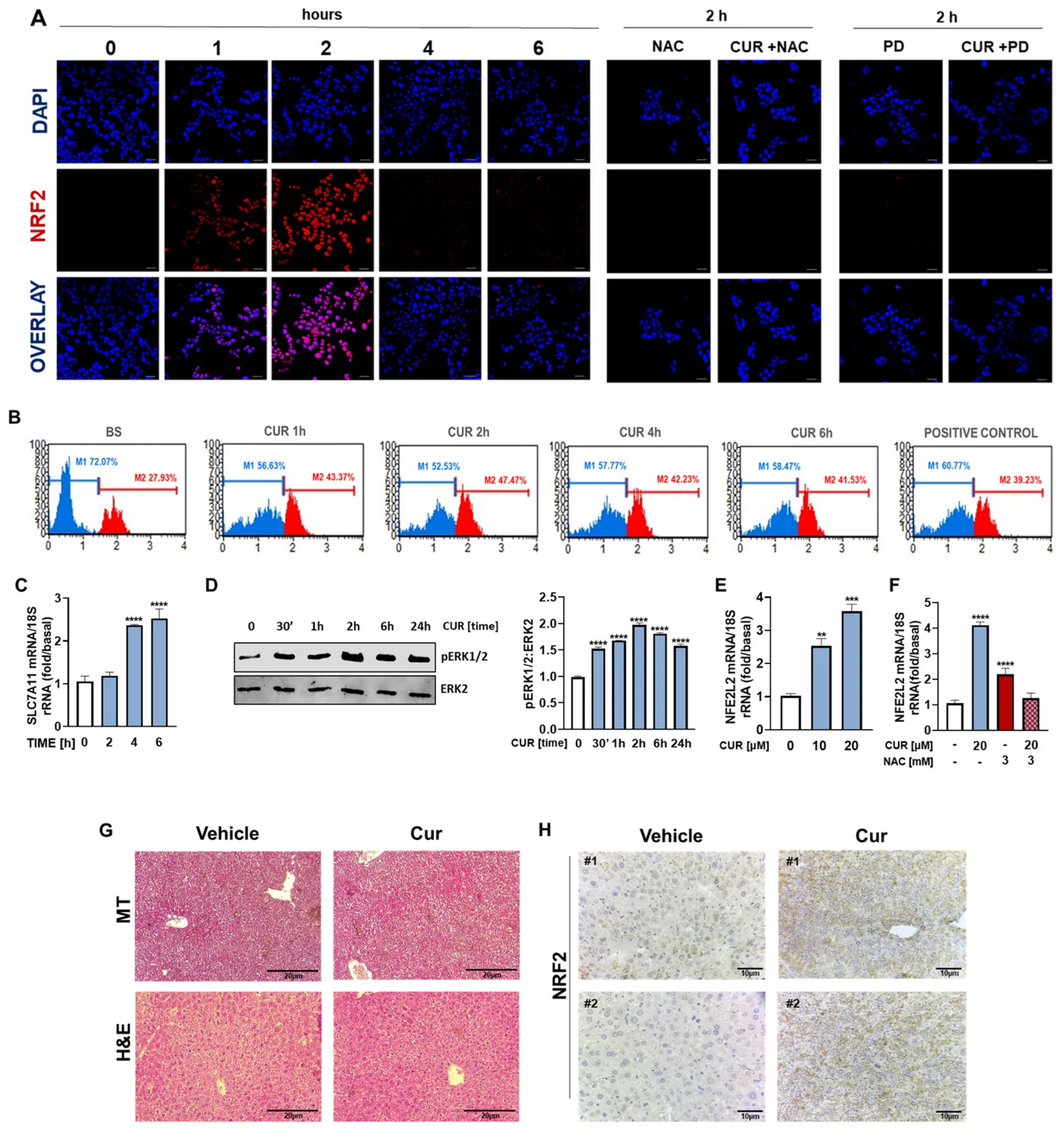
NRF2 guides an antioxidant response in CUR-treated H295R cells. (**A**) Analysis by immunofluorescence of NRF2 in H295R cells treated with curcumin (20 μm), PD98059 (PD) (10 μm), or N-acetylcysteine (NAC) (3 mM), as indicated. Scale bar: 50 µm. (**B**) Flow cytometric analysis of reactive oxygen species (ROS) in H295R cells treated with curcumin 20 μm for indicated times. Cells treated with H_2_O_2_ for 2 h were used as a positive control. ROS-positive cells are shown in red. (**C**) mRNA expression of *SLC7A11* in H295R cells treated with curcumin 20 μm for the indicated times. Graphs are the means ± SD of results from three independent experiments. **** *p* < 0.0001. (**D**) Time-course activation of pERK1/2 in H295R cells treated with curcumin (20 μM). Graph represents densitometric analysis of pERK1/2 normalized to ERK2. Graphs are the means ± SD of results from three independent experiments. **** *p* < 0.0001. (**E**,**F**) mRNA expression of *NFE2L2* (*NRF2* gene) in H295R cells treated for 48 h with the indicated concentrations of curcumin and NAC. Graphs are the means ± SD of results from three independent experiments. ** *p* < 0.01; *** *p* < 0.001; **** *p* < 0.0001. (**G**) Masson’s trichrome (MT) staining and hematoxylin and eosin (H&E) staining of liver tissues. Scale bar: 20 µm. (**H**) Immunohistochemical (IHC) analysis for NRF2 performed on liver tissues. Scale bar: 10 µm.

To identify transcription factors upregulated in the ROS-rich environment induced by curcumin, we analyzed the expression of NRF2 (from *NFE2L2* gene), which acts as the master regulator of the cellular antioxidant response [17]. Under basal conditions, NRF2 is retained in the cytoplasm through its interaction with KEAP1. Upon phosphorylation, KEAP1 undergoes rapid degradation, allowing NRF2 to translocate into the nucleus, where it upregulates the transcription of target genes, including *SLC7A11*. To assess this pathway, we monitored the time-dependent subcellular localization of NRF2 following curcumin treatment, which revealed peak nuclear accumulation after 2 h (Figure 4A), consistent with previous findings in other cell models [18]. This rapid response prompted us to investigate whether intracellular ROS production occurred with similar early kinetics. A time-course experiment demonstrated a significant increase in ROS levels as early as 1 h (Figure 4B), suggesting that oxidative stress precedes maximal NRF2 nuclear translocation. Notably, scavenging ROS with NAC completely abrogated the effects of the compound on NRF2 signaling (Figure 4A). Regarding downstream targets, *SLC7A11* mRNA expression peaked at 4 h (Figure 4C), showing a temporal lag compared to the initial transcription factor activation. This delay provides strong mechanistic evidence of a canonical stress response, confirming that curcumin-driven *SLC7A11* upregulation is sequentially mediated by NRF2.

NRF2 activity and nuclear translocation are well known to be regulated by the ERK pathway, which was previously shown to be activated by curcumin in H295R cells [19]. Our Western blot data confirmed a rapid and sustained increase in ERK phosphorylation in response to curcumin (Figure 4D). The use of PD98059 prevented NRF2 activation (Figure 4A), confirming that curcumin effects are elicited through a ROS-ERK-NRF2-SLC7A11 axis.

A sustained activation of this antioxidant pathway in response to curcumin is supported by the observations that *NRF2* mRNA is still upregulated after 48 h of treatment (Figure 4E), and the use of NAC abrogated curcumin effects on *NRF2* transcription (Figure 4F).

To evaluate the in vivo safety profile of curcumin, histological and immunohistochemical (IHC) analyses were performed on liver tissues from mice supplemented with curcumin (500 mg/kg for 30 days). Masson’s trichrome (MT) staining and hematoxylin and heosin (H&E) staining revealed no signs of fibrosis, collagen deposition, or structural remodeling in the liver parenchyma of curcumin-treated mice compared to vehicle-treated controls (Figure 4G), confirming the absence of hepatotoxicity. Concurrently, IHC analysis demonstrated a marked increase in Nrf2 nuclear translocation within the healthy liver tissue of treated animals (Figure 4H). Together, these findings indicate that curcumin safely stimulates the Nrf2-mediated antioxidant response in vivo without inducing structural or morphological alterations in the liver.

**Figure 5:**
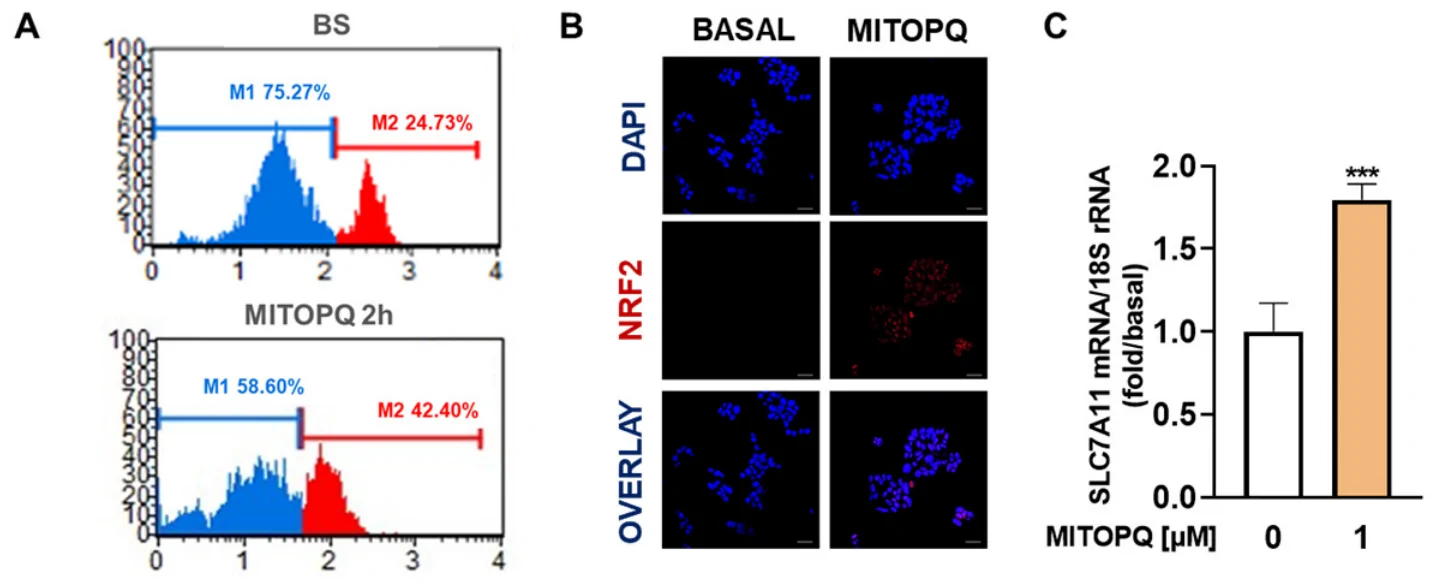
Mitochondrial oxidative stress drives a generalizable NRF2/SLC7A11 antioxidant cascade in ACC cells. (**A**) Flow cytometric analysis of reactive oxygen species (ROS) in ACC cells treated with MitoPQ (1 μM) for the indicated times. (**B**) Analysis by immunofluorescence of NRF2 expression in H295R cells treated with MitoPQ for 4 h at the indicated times. Scale bar: 50 µm. (**C**) mRNA expression of SLC7A11 in H295R cells treated for 6 h with MitoPQ (1 μM). Graphs are the means ± SD of results from three independent experiments. *** *p* < 0.001.

To establish NRF2 activation as a general mechanism elicited by ROS in ACC cells, we introduced a gain-of-function approach utilizing MitoPQ, a selective inducer of mitochondrial ROS production via complex I inhibition. Remarkably, a brief incubation with MitoPQ fully recapitulated the molecular kinetics observed in cells treated with curcumin. Specifically, treatment triggered a rapid elevation in intracellular ROS levels within 2 h (Figure 5A), which subsequently promoted the enhanced nuclear translocation of NRF2 at 4 h (Figure 5B), culminating in a significant upregulation of *SLC7A11* mRNA expression at 6 h (Figure 5C). Collectively, the strict temporal sequence of these events, combined with the identical cellular response elicited by a direct mitochondrial ROS trigger, provides robust and independent evidence positioning NRF2 as the central driver of the antioxidant response in ACC.

### 3.5. Curcumin Potentiates Effects of Sub-Therapeutic Doses of Mitotane

**Figure 6:**
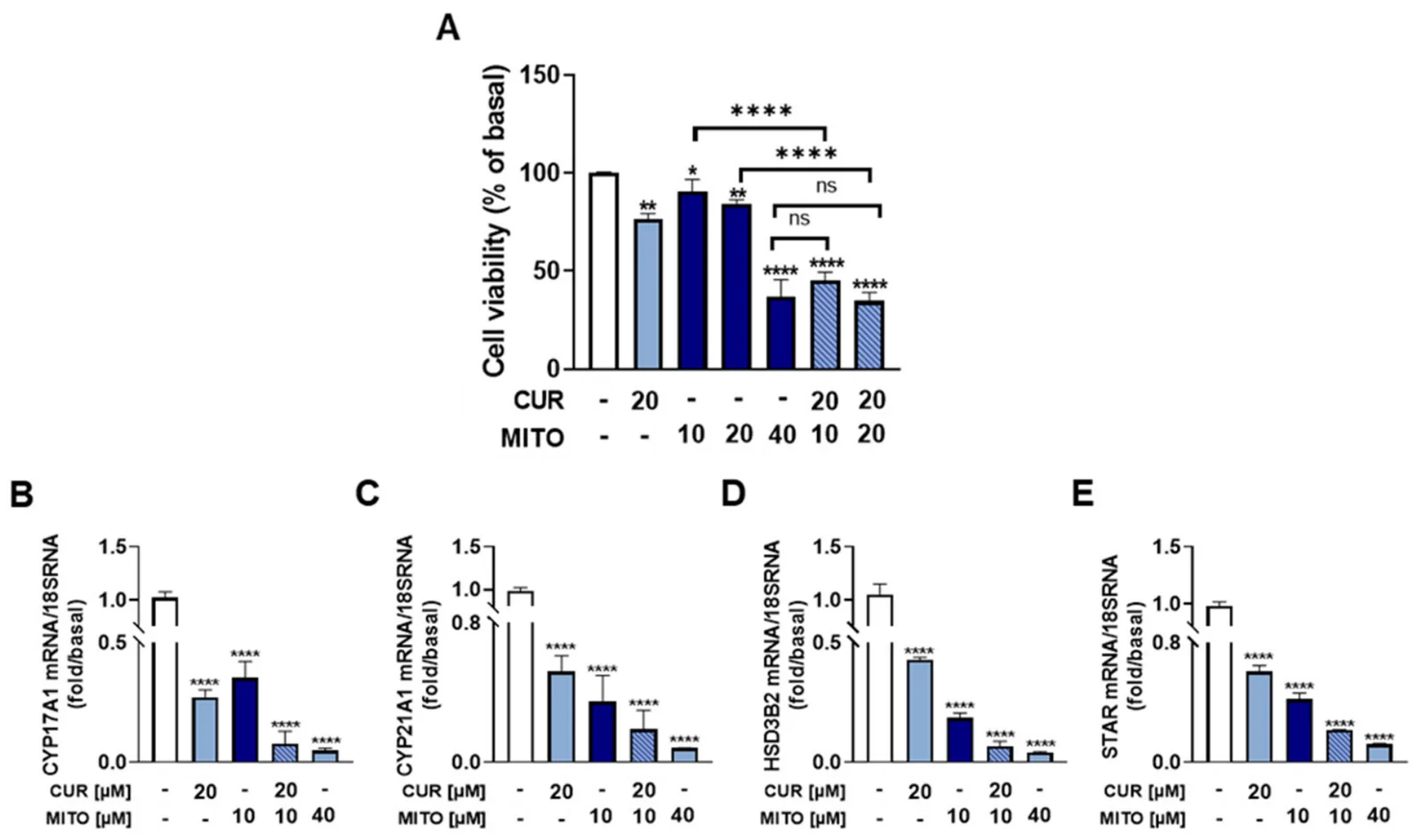
Curcumin potentiates effects of sub-therapeutic effects of mitotane. (**A**) H295R cells treated for 48 h with the indicated doses of curcumin and mitotane. Graph represents the means ± SD of three independent experiments. * *p* < 0.05; ** *p* < 0.01; **** *p* < 0.0001; ns: not significant). (**B**–**E**) mRNA expression of genes related to steroid synthesis in H295R cells treated for 48 h with curcumin and mitotane as indicated. Bars represent means ± SD of data from three independent RNA samples. **** *p* < 0.0001.

Achieving therapeutic plasma concentrations of mitotane remains a major clinical challenge, as more than 50% of patients fail to reach the recommended therapeutic range of 14–20 mg/L (corresponding to 40–60 μM) [20]. We therefore tested whether combining curcumin with sub-therapeutic doses of mitotane could enhance antitumor efficacy and potentially provide clinical benefits. Specifically, we used the doses of 10 μM and 20 μM, corresponding to plasma concentrations of approximately 3 and 6.5 mg/mL, respectively. The MTT assay demonstrated that while both drugs individually decreased cell viability, the inhibitory effect was significantly enhanced by the combination (Figure 6A). Drug interaction analyses revealed a strong synergistic action between cur and mitotane, with the highest single-agent (HSA) scores ≥ 20. This synergism was further corroborated by multiple computational models including Loewe, Bliss, and ZIP (Figure S1). Combining curcumin with sub-therapeutic doses of mitotane inhibited cell proliferation to a degree comparable to the 40 μM therapeutic dose of mitotane alone (Figure 6A). Importantly, the combination of 10 μM mitotane and 20 μM curcumin exerted a stronger inhibitory effect on the mRNA expression of the steroidogenic enzymes *StAR*, *CYP17A1*, *HSD3B2*, and *CYP21A1* compared to either monotherapy. Furthermore, these effects fully overlapped with those induced by the therapeutic dose of 40 μM mitotane (Figure 6B–E).

## 4. Discussion

Achieving therapeutic plasma concentration of mitotane (approximately 14 mg/L, equivalent to 40 μM) is notoriously challenging, and patients with adrenocortical carcinoma (ACC) frequently experience severe side effects that lead to drug discontinuation [20]. Developing novel approaches to mitigate therapy-related toxicity and improve clinical outcomes remains an unexplored frontier in ACC management. In this context, nutraceuticals represent promising agents currently being evaluated as adjuvant treatments for several diseases, including cancer. These bioactive compounds are capable of modulating various hallmark pathways involved in carcinogenesis, such as cell proliferation, cell death, angiogenesis, inflammation, and immune surveillance [21].

Curcuma longa has gained worldwide scientific attention for its diverse biological activities, including its anticancer potential [4], mediated by the activation of distinct cell death mechanisms [5]. However, its clinical application is limited by poor oral bioavailability, rapid metabolism, and fast systemic clearance. Although standard high-dose oral supplementation results in maximum plasma levels of only 0.5–2 μM, specialized delivery systems can enhance these concentrations by 10- to 40-fold [4]. Therefore, it is reasonable to postulate that the 10 and 20 µM doses used in this study could be clinically achieved in humans through the daily supplementation of advanced curcumin formulations. Notably, we have developed a bioavailability-enhanced lipid-polymeric nanoformulation of curcumin (CUR-LIPOMER), which demonstrated a 4-fold increased efficacy in ACC cells (manuscript in preparation). Additionally, Signpath Pharma has developed LipoCurc™, a proprietary intravenous liposomal formulation that achieves sustained active levels of curcumin over 1000-fold greater than traditional oral dosing. Crucially, LipoCurc™ has successfully completed Phase 1a and 1b clinical trials in human subjects (NCT01403545, NCT02138955), demonstrating excellent tolerability with no toxicity and showing early signs of efficacy even in end-stage, heavily pretreated cancer patients. Reflecting its therapeutic promise, this liposomal agent is currently recruiting patients for a Phase II clinical study (NCT05768919). Although we previously demonstrated that curcumin impairs ACC growth [6], a more comprehensive understanding of its underlying molecular mechanisms is required to establish its efficacy as a clinical adjuvant. Expanding upon our prior finding that curcumin alters ACC cell metabolism by reducing cholesterol availability while promoting glucose and glutamine consumption [6], the present study aimed to uncover the regulatory networks driving these metabolic rearrangements.

Cholesterol can be synthesized de novo through the mevalonate pathway, a metabolic route that utilizes acetyl-CoA as the primary substrate. This pathway culminates in the production of isopentenyl pyrophosphate (IPP), a key isoprenoid building block, which is further modified through sequential enzymatic steps to yield cholesterol. In healthy cells, cholesterol amounts are finely tuned through a multi-tiered regulatory network that includes transcriptional control. The primary transcription factors are Sterol Responsive Element Binding Protein 1 and 2 (*SREBP1/2*), alongside SF-1 in adrenal cells [8]. All three transcription factors were downregulated by curcumin in ACC cells, resulting in a concomitant decrease in the expression of key cholesterol biosynthesis genes. It is worth noting that these effects were also elicited in MUC-1 cells, a mitotane- and chemotherapy-resistant ACC model [13]. Given that SF-1 is critical for the transcription of steroidogenic enzymes [22], we also observed a consistent reduction in their expression. This finding is highly relevant for ACC patients, as the therapeutic actions of mitotane rely on both its cytotoxic effects and its capacity to reduce steroid production. Clinically, a vast majority of patients present with symptoms of hormone overproduction, primarily cortisol. Crucially, cortisol-secreting tumors carry a significantly worse prognosis, reduced survival, and elevated recurrence risk compared to their non-secreting counterparts [23].

To elucidate the mechanism underlying curcumin’s ability to upregulate glycolytic genes, we investigated its effect on the transcription factor Hypoxia Inducible Factor-1 alpha (*HIF1α*), a well-established driver of glycolysis that sustains cancer cell growth and survival [24]. As anticipated, curcumin treatment induced *HIF1α* expression. This increase was dependent on sustained ROS production, as demonstrated by the ability of NAC to reverse the effects of curcumin on both *HIF1α* and its downstream targets. Consistently, numerous studies have reported that the anticancer mechanisms of curcumin are largely mediated by induction of oxidative stress [25,26,27]. Indeed, curcumin can directly stimulate ROS production [28] or potentiate ROS generation induced by other agents [29,30,31]. This pro-oxidant activity occurs despite its conventional antioxidant capacity, which stems from its chemical structure and ability to act as a radical-trapping molecule. Indeed, we showed that in our system, curcumin has a dual action; increases ROS and potentiates an adaptive antioxidant response. We established that glutamine influx through the SLC1A5 transporter is required to support intracellular glutamate levels derived from GLS1 activity. This glutamate, alongside cysteine (via SLC7A11) and glycine (via SLC6A9), serves as the necessary precursor for glutathione (GSH) biosynthesis. This antioxidant mechanism explains the absence of phospholipid peroxidation in our model. In contrast, in other cancer phenotypes, curcumin has been shown to suppress tumorigenesis by inducing ferroptosis—a form of regulated cell death strictly dependent on lipid peroxidation [32,33].

The transcription factor NRF2 (from NFE2L2 gene) acts as the master regulator of cellular antioxidant response [17]. NRF2 activation increased following curcumin treatment and was dependent on ROS production and ERK activation. In fact, the use of the ROS scavenger NAC and ERK inhibitor PD98059 prevented NRF2 activation. Despite this cellular attempt to counteract oxidative stress, curcumin-treated ACC cells ultimately undergo apoptotic cell death [6]. Indeed, the same regulatory mechanism has been described in curcumin-treated gastric cancer cells, where ERK phosphorylation correlates with mitochondrial damage, increased ROS production, and cytochrome c release, thereby triggering the intrinsic apoptotic pathway [34]. Interestingly, NAC treatment alone caused an increase in *NRF2* mRNA expression. This is a well-documented phenomenon that aligns with NRF2 established role in modulating cellular redox-sensitive signaling pathways [35,36]. Under baseline conditions (NAC alone), the lack of pro-oxidant stimuli leaves the Keap1 sensing machinery fully intact and operational. Consequently, any newly translated NRF2 protein is rapidly sequestered in the cytoplasm, preventing its nuclear translocation. Accordingly, we found that NRF2 activation did not occur with NAC alone, and the NRF2 target gene, *SLC7A11*, was not upregulated. This definitively proves that ROS generation uniquely driven by curcumin is the indispensable master switch required to inactivate Keap1, thereby stabilizing the NRF2 protein and allowing it to transactivate the *SLC7A11* promoter. Furthermore, by using MitoPQ—a selective inducer of mitochondrial complex I ROS—we were able to induce NRF2 nuclear translocation and subsequent *SLC7A11* upregulation, underscoring the high reproducibility of the ROS/NRF2/SLC7A11 axis. These complementary lines of evidence firmly establish NRF2 as a master regulator of the antioxidant response in ACC.

Because the inhibitory effects induced by curcumin partially mirror those reported for mitotane, we investigated whether a co-treatment strategy could enhance anti-tumor efficacy. Our findings indicate that the combination of curcumin and sub-therapeutic doses of mitotane exerts a synergistic inhibitory effect on cell viability, achieving an outcome comparable to a full therapeutic dose. Concurrently, a synergistic suppression of steroidogenic enzyme transcription was noted.

## 5. Conclusions

In summary, this study delineates the multifaceted mechanism of action of curcumin in ACC cells, which involves triggering ROS-induced apoptosis alongside a simultaneous, compensatory antioxidant response. Crucially, our key finding is the robust synergy between curcumin and mitotane. This interaction enables sub-therapeutic doses of mitotane to inhibit cell proliferation and steroid hormone expression, with an efficacy equivalent to that of the therapeutic dose.

Based on these data, curcumin may hold potential as an adjuvant approach for ACC management, particularly for those patients who fail to achieve or maintain target therapeutic plasma concentrations of mitotane. Crucially, while our current in vivo model was limited to curcumin monotherapy in tumor-free mice, these findings support the hypothesis that the curcumin–mitotane combination may warrant further evaluation in tumor-bearing animal models to validate its efficacy, pharmacokinetics, and safety prior to any clinical application.

## Data Availability

The original contributions presented in this study are included in the article/Supplementary Material. Further inquiries can be directed to the corresponding authors.

## Supplementary Materials

The following supporting information can be downloaded at: https://www.mdpi.com/article/10.3390/cells15161498/s1, Figure S1: Synergy analysis of the combined treatment with curcumin and mitotane in H295R cells. Synergy between curcumin and mitotane was evaluated using SynergyFinder+ based on four reference models: Bliss (A), Highest Single Agent (HSA) (B), Loewe additivity (C), and Zero Interaction Potency (ZIP) (D). Positive synergy scores indicate a synergistic interaction between the two compounds, whereas scores around zero indicate an additive effect and negative scores indicate antagonism. The mean synergy score and corresponding *p*-value are reported for each model. The HSA model was considered the primary reference model for the assessment of drug interaction. (E) Dose–response matrix showing the percentage of cell growth inhibition obtained with the indicated concentrations of curcumin and mitotane. Curcumin was tested at 0, 10, and 20 μM and mitotane at 0, 10, and 20 μM.

## Abbreviations

The following abbreviations are used in this manuscript:

ACC	adrenocortical carcinoma
CYP11A1	Cytochrome P450 Family 11 Subfamily A Member 1
CYP17A1	Cytochrome P450 Family 17 Subfamily A Member 1
DHCR7	7-dehydrocholesterol reductase
ERK1/2	Extracellular signal-Regulated Kinase1/2
GLS1	Glutaminase 1
GSS	Glutathione synthase
H&E	hematoxylin-eosin staining
HIF1α	Hypoxia inducible factor
HK1	hexokinase-1
HK2	hexokinase-2
HMGCR	3-hydroxy-3-methylglutaryl-coenzyme A reductase
HSD3B2	3beta-hydroxysteroid dehydrogenase/delta(5)-delta(4)isomerase type II
MitoPQ	MitoParaquat
MT	Masson’s Trichrome staining
MVK	mevalonate kinase
NAC	N-acetylcysteine
NRF2	Nuclear factor erythroid 2-related factor 2
ROS	reactive oxygen species
SF-1	Steroidogenic Factor-1
SLC1A5	Solute Carrier Family 1 Member 5
SLC6A9	Solute Carrier Family 6 Member 9
SLC7A11	Solute Carrier Family 7 Member 11
SQLE	Squalene epoxidase
SREBP1	Sterol responsive element binding protein-1
SREBP2	Sterol responsive element binding protein-2
StAR	Steroidogenic Acute Regulatory protein

## Appendix A

**Table A1:** QPCR primer sequence.

Gene Name	Forward Primer 5′–3′	Reverse Primer 5′–3′
*18S*	CGGCGACGACCCATTCGAAC	GAATCGAACCCTGATTCCCCGTC
*CYP11A1*	TCCAGAAGTATGGCCCGATT	CATCTTCAGGGTCGATGACATAAA
*CYP17A1*	TCTCTGGGCGGCCTCAA	AGGCGATACCCTTACGGTTGT
*CYP21A2*	TCAGGTTCTTCCCCAATCCA	TCCACGATGTGATCCCTCTTC
*DHCR7*	GCAGGGGTTGTGAACAAGTAT	GAGACGGCATAGCCAAGGAT
*GLS1*	TCCTCAACTGGCCAAATTC	CAGAAGGGAACTTTGGTATCTC
*GSS*	GCCATAGAGAATGAGCTACTG	AAACAGCCTTCGGTCTTG
*HIF-1α*	AGCCGAGGAAGAACTATGA	CACACTGAGGTTGGTTACTG
*HK1*	CAACAGCCACAGTCAAGAT	AAGGAAGACCCACCAAGA
*HK2*	GAGTGGAGATGCACAACAA	CTGGACAATGTGGTCAAAGA
*HMGCR*	GTCATTCCAGCCAAGGTTGT	GGGACCACTTGCTTCCATTA
*HSD3B2*	GCGGCTAATGGGTGGAATCTA	CATTGTTGTTCAGGGCCTCAT
*MVK*	TCCGATACCATCAAGGGAAGA	TTTGGTGTTGGTCAGCAGGAT
*NR5A1 (SF-1)*	GGAGTTTGTCTGCCTCAAGTTCA	CGTCTTTCACCAGGATGTGGTT
*NFE2L2 (NRF2)*	TGCCCACATTCCCAAATC	CTGAAACGTAGCCGAAGAAA
*SLC1A5*	TGCCTTTGGGACCTCTT	TTGGCCACGCCATTATTC
*SLC6A9*	TCACCATGGCTTCCTACA	CCGAGGATGGAGAAGATGA
*SLC7A11*	CGACCATTAATGCTGAGGAG	AGGAGAGGGCAACAAAGA
*SQLE*	GCTTCCTTCCTCCTTCATC	GTCATTCCTCCACCAGTAAG
*SREBP1*	CGGAACCATCTTGGCAACA	GCCGGTTGATAGGCAGCTT
*SREBP2*	GAAGCCCTCTATTGGATGATG	AGGTGAGGACACACAGAA
*StAR*	CCACCCCTAGCACGTGGA	TCCTGGTCACTGTAGAGAGTCTCTTC

## References

[1] Fassnacht M., Assie G., Baudin E., Eisenhofer G., de la Fouchardiere C., Haak H.R., de Krijger R., Porpiglia F., Terzolo M., Berruti A. (2020). Adrenocortical carcinomas and malignant phaeochromocytomas: ESMO–EURACAN Clinical Practice Guidelines for diagnosis, treatment and follow-up. Ann. Oncol..

[2] Fassnacht M., Puglisi S., Kimpel O., Terzolo M. (2025). Adrenocortical carcinoma: A practical guide for clinicians. Lancet Diabetes Endocrinol..

[3] Mercier B.D., Tizpa E., Philip E.J., Feng Q., Huang Z., Thomas R.M., Pal S.K., Dorff T.B., Li Y.R. (2022). Dietary Interventions in Cancer Treatment and Response: A Comprehensive Review. Cancers.

[4] Nocito M.C., De Luca A., Prestia F., Avena P., La Padula D., Zavaglia L., Sirianni R., Casaburi I., Puoci F., Chimento A. (2021). Antitumoral Activities of Curcumin and Recent Advances to ImProve Its Oral Bioavailability. Biomedicines.

[5] Yu C., Yang B., Najafi M. (2021). Targeting of cancer cell death mechanisms by curcumin: Implications to cancer therapy. Basic Clin. Pharmacol. Toxicol..

[6] Nocito M.C., Avena P., Zavaglia L., De Luca A., Chimento A., Hamad T., La Padula D., Stancati D., Hantel C., Sirianni R. (2023). Adrenocortical Carcinoma (ACC) Cells Rewire Their Metabolism to Overcome Curcumin Antitumoral Effects Opening a Window of Opportunity to Improve Treatment. Cancers.

[7] Trotta F., Avena P., Chimento A., Rago V., De Luca A., Sculco S., Nocito M.C., Malivindi R., Fallo F., Pezzani R. (2020). Statins Reduce Intratumor Cholesterol Affecting Adrenocortical Cancer Growth. Mol. Cancer Ther..

[8] Baba T., Otake H., Inoue M., Sato T., Ishihara Y., Moon J.Y., Tsuchiya M., Miyabayashi K., Ogawa H., Shima Y. (2018). Ad4BP/SF-1 regulates cholesterol synthesis to boost the production of steroids. Commun. Biol..

[9] Parker K.L., Schimmer B.P. (1993). Transcriptional regulation of the adrenal steroidogenic enzymes. Trends Endocrinol. Metab..

[10] Baba T., Otake H., Sato T., Miyabayashi K., Shishido Y., Wang C.Y., Shima Y., Kimura H., Yagi M., Ishihara Y. (2014). Glycolytic genes are targets of the nuclear receptor Ad4BP/SF-1. Nat. Commun..

[11] Sbiera S., Schmull S., Assie G., Voelker H.U., Kraus L., Beyer M., Ragazzon B., Beuschlein F., Willenberg H.S., Hahner S. (2010). High Diagnostic and Prognostic Value of Steroidogenic Factor-1 Expression in Adrenal Tumors. J. Clin. Endocrinol. Metab..

[12] Li X., Wang Y., Xiong M., Xie C., Yang D. (2025). The therapeutic effect of curcumin in metabolic dysfunction-associated steatotic liver disease: A systematic review and meta-analysis of animal studies. Front. Pharmacol..

[13] Hantel C., Shapiro I., Poli G., Chiapponi C., Bidlingmaier M., Reincke M., Luconi M., Jung S., Beuschlein F., Hantel C. (2016). Targeting heterogeneity of adrenocortical carcinoma: Evaluation and extension of preclinical tumor models to improve clinical translation. Oncotarget.

[14] Tang J. (2017). Informatics approaches for predicting, understanding, and testing cancer drug combinations. Methods Mol. Biol..

[15] Islam M.A., Kumar S. (2026). Masson’s Trichrome Staining Technique to Evaluate Tissue Fibrosis. Methods Mol. Biol..

[16] Biswas S.K., McClure D., Jimenez L.A., Megson I.L., Rahman I. (2004). Curcumin Induces Glutathione Biosynthesis and Inhibits NF-κB Activation and Interleukin-8 Release in Alveolar Epithelial Cells: Mechanism of Free Radical Scavenging. Antioxid. Redox Signal..

[17] Chen W.T., McKee N.W., Kuhnell D., Dodson M. (2026). NRF2: Master regulator of cellular homeostasis and therapeutic vulnerability in cancer. Redox Biol..

[18] Hu Y., Cheng L., Du S., Wang K., Liu S. (2024). Antioxidant curcumin induces oxidative stress to kill tumor cells (Review). Oncol. Lett..

[19] Huang X., Liang C., Yang H., Li X., Deng X., Liang X., Li L., Huang Z., Lu D., Ma Y. (2021). Curcumin induces apoptosis and inhibits the growth of adrenocortical carcinoma: Identification of potential candidate genes and pathways by transcriptome analysis. Oncol. Lett..

[20] Fassnacht M., Dekkers O.M., Else T., Baudin E., Berruti A., De Krijger R.R., Haak H.R., Mihai R., Assie G., Terzolo M. (2018). European Society of Endocrinology Clinical Practice Guidelines on the management of adrenocortical carcinoma in adults, in collaboration with the European Network for the Study of Adrenal Tumors. Eur. J. Endocrinol..

[21] Altomare C., Macrì R., Serra M., Ussia S., Ritorto G., Maiuolo J., Muscoli C., Perri E., Mollace V. (2025). The Potential of Nutraceutical Supplementation in Counteracting Cancer Development and Progression: A Pathophysiological Perspective. Nutrients.

[22] Ikeda Y., Shen W.H., Ingraham H.A., Parker K.L. (1994). Developmental expression of mouse steroidogenic factor-1, an essential regulator of the steroid hydroxylases. Mol. Endocrinol..

[23] Berruti A., Fassnacht M., Haak H., Else T., Baudin E., Sperone P., Kroiss M., Kerkhofs T., Williams A.R., Ardito A. (2014). Prognostic Role of Overt Hypercortisolism in Completely Operated Patients with Adrenocortical Cancer. Eur. Urol..

[24] Gordan J.D., Thompson C.B., Simon M.C. (2007). HIF and c-Myc: Sibling Rivals for Control of Cancer Cell Metabolism and Proliferation. Cancer Cell.

[25] Simon H.U., Haj-Yehia A., Levi-Schaffer F. (2000). Role of reactive oxygen species (ROS) in apoptosis induction. Apoptosis.

[26] Gabr S.A., Elsaed W.M., Eladl M.A., El-Sherbiny M., Ebrahim H.A., Asseri S.M., Eltahir Y.A.M., Elsherbiny N., Eldesoqui M. (2022). Curcumin Modulates Oxidative Stress, Fibrosis, and Apoptosis in Drug-Resistant Cancer Cell Lines. Life.

[27] Lin X., Wang L., Zhao L., Zhu Z., Chen T., Chen S., Tao Y., Zeng T., Zhong Y., Sun H. (2020). Curcumin micelles suppress gastric tumor cell growth by upregulating ROS generation, disrupting redox equilibrium and affecting mitochondrial bioenergetics. Food Funct..

[28] Chan W.H., Wu H.Y., Chang W.H. (2006). Dosage effects of curcumin on cell death types in a human osteoblast cell line. Food Chem. Toxicol..

[29] Sánchez Y., Simón G.P., Calviño E., De Blas E., Aller P. (2010). Curcumin Stimulates Reactive Oxygen Species Production and Potentiates Apoptosis Induction by the Antitumor Drugs Arsenic Trioxide and Lonidamine in Human Myeloid Leukemia Cell Lines. J. Pharmacol. Exp. Ther..

[30] Lee W.H., Loo C.Y., Traini D., Young P.M. (2020). Development and Evaluation of Paclitaxel and Curcumin Dry Powder for Inhalation Lung Cancer Treatment. Pharmaceutics.

[31] Zhang N., Gao M., Wang Z., Zhang J., Cui W., Li J., Zhu X., Zhang H., Yang D.H., Xu X. (2021). Curcumin reverses doxorubicin resistance in colon cancer cells at the metabolic level. J. Pharm. Biomed. Anal..

[32] Jiang X., Stockwell B.R., Conrad M. (2021). Ferroptosis: Mechanisms, biology and role in disease. Nat. Rev. Mol. Cell Biol..

[33] Tang X., Ding H., Liang M., Chen X., Yan Y., Wan N., Chen Q., Zhang J., Cao J. (2021). Curcumin induces ferroptosis in non-small-cell lung cancer via activating autophagy. Thorac. Cancer.

[34] Cao A.L., Tang Q.F., Zhou W.C., Qiu Y.Y., Hu S.J., Yin P.H. (2015). Ras/ERK signaling pathway is involved in curcumin-induced cell cycle arrest and apoptosis in human gastric carcinoma AGS cells. J. Asian Nat. Prod. Res..

[35] Jannatifar R., Parivar K., Roodbari N.H., Nasr-Esfahani M.H. (2020). The Effect of N-Acetyl-Cysteine on NRF 2 Antioxidant Gene Expression in Asthenoteratozoospermia Men: A Clinical Trial Study. Int. J. Fertil. Steril..

[36] Bottoni L., Minetti A., Realini G., Pio E., Giustarini D., Rossi R., Rocchio C., Franci L., Salvini L., Catona O. (2024). NRF2 activation by cysteine as a survival mechanism for triple-negative breast cancer cells. Oncogene.

